# Modified RNAs and predictions with the ViennaRNA Package

**DOI:** 10.1093/bioinformatics/btad696

**Published:** 2023-11-16

**Authors:** Yuliia Varenyk, Thomas Spicher, Ivo L Hofacker, Ronny Lorenz

**Affiliations:** Department of Theoretical Chemistry, University of Vienna, Vienna 1090, Austria; Vienna BioCenter PhD Program, Doctoral School of the University of Vienna and Medical University of Vienna, Vienna 1030, Austria; Department of Theoretical Chemistry, University of Vienna, Vienna 1090, Austria; UniVie Doctoral School Computer Science (DoCS), University of Vienna, Vienna 1090, Austria; Department of Theoretical Chemistry, University of Vienna, Vienna 1090, Austria; Research Group Bioinformatics and Computational Biology, Faculty of Computer Science, University of Vienna, Vienna 1090, Austria; Department of Theoretical Chemistry, University of Vienna, Vienna 1090, Austria

## Abstract

**Motivation:**

In living organisms, many RNA molecules are modified post-transcriptionally. This turns the widely known four-letter RNA alphabet ACGU into a much larger one with currently more than 300 known distinct modified bases. The roles for the majority of modified bases remain uncertain, but many are already well-known for their ability to influence the preferred structures that an RNA may adopt. In fact, tRNAs sometimes require certain modifications to fold into their cloverleaf shaped structure. However, predicting the structure of RNAs with base modifications is still difficult due to the lack of efficient algorithms that can deal with the extended sequence alphabet, as well as missing parameter sets that account for the changes in stability induced by the modified bases.

**Results:**

We present an approach to include sparse energy parameter data for modified bases into the ViennaRNA Package. Our method does not require any changes to the underlying efficient algorithms but instead uses a set of plug-in constraints that adapt the predictions in terms of loop evaluation at runtime. These adaptations are efficient in the sense that they are only performed for loops where additional parameters are actually available for. In addition, our approach also facilitates the inclusion of more modified bases as soon as further parameters become available.

**Availability and implementation:**

Source code and documentation are available at https://www.tbi.univie.ac.at/RNA.

## 1 Introduction

Chemical modifications are highly abundant in different types of RNA sequences and serve a variety of functions (Sloan *et al.* 2016, Lorenz *et al.* 2017, Boo and Kim 2020). At the time of writing, the Modomics database (Boccaletto *et al.* 2022) lists more than 300 known modifications of RNA residues. These modifications affect RNA structure (Tanzer *et al.* 2019), cell regulation (Delaunay and Frye 2019, Wilkinson *et al.* 2022), splicing regulation (Wang *et al.* 2022), binding (Boo and Kim 2020), and other functions (Jonkhout *et al.* 2017, Nachtergaele and He 2018). Understanding the RNA sequence’s structure is crucial to comprehend the structure–function relationship (Vandivier *et al.* 2016). Therefore, accurate prediction of the secondary structure is of great interest in RNA research.

Physics-based secondary structure prediction algorithms, such as implemented in the ViennaRNA Package (Lorenz *et al.* 2011) and RNAstructure (Reuter and Mathews 2010), utilize the nearest-neighbor model that employs Turner energy parameters (Turner and Mathews 2009). However, due the lack of thermodynamical data on parameters for modified bases, available prediction software typically restrains itself to the ACGU alphabet, with only very few exceptions (Tanzer *et al.* 2019, Kierzek *et al.* 2022). In recent years, several studies provided information for some modifications, making it possible to expand the standard four-letter alphabet and improve the secondary structure prediction for sequences containing those particular modified bases.

To address this problem we introduced modified base support in the ViennaRNA Package starting with version 2.6.0. The changes applied to our implementation include a set of currently available energy parameters for modifications and the means to incorporate this data into the prediction algorithms. Additionally, we developed an accessible and user-friendly approach to specify and include more parameters for additional modified bases that have yet to be investigated.

## 2 Methods

### 2.1 Modified base energy corrections

In principle, structure prediction including modified bases is straightforward: (i) the implemented prediction algorithm must be able to operate on an extended nucleotide alphabet and (ii) distinguish the additional base pairs that involve one or two modified bases. Such adaptations to existing implementations are not expected to substantially change the algorithm’s runtime. However, parameters for the Nearest Neighbor (NN) energy model are usually stored within look-up tables as multi-dimensional matrices, where each dimension corresponds to one of the delimiters of a loop, e.g. one or two base pairs or a number of individual bases. It is fairly easy to see that each modified base that is additionally taken into account by a prediction algorithm has a large impact on the growth of the NN energy tables, see also Supplementary Section S1.4.

Free energy parameters for loops composed of the canonical nucleotides ACGU are available for any type of nested loop (Turner and Mathews 2009), but only a very small subset of parameters exists for loops that contain modified bases. In particular, most available energy parameters for loops with modified bases are restricted to stacked base pairs. Recently, Kierzek *et al.* (2022) used a set of *m*^6^*A* parameters that mostly consists of stacked pairs and a few terminal mismatches to compile a complete set of all NN loop parameters. Here, the authors simply replaced any missing loop parameters by their unmodified counterpart adenosine instead of *m*^6^*A*.

In contrast, our new implementation within the ViennaRNA Package does not require a pre-compiled *complete* set of NN energy parameters with modified bases. Instead, we utilize *hard-* and *soft-constraints* (Lorenz *et al.* 2016) to modify the predictions only for the subset of loops where NN parameters are actually available for. Missing loop parameters are implicitly replaced by those of a *fallback* base, typically the unmodified counterpart. More precisely, our underlying prediction algorithms do not require any changes in implementation with respect to the nucleotide alphabet or set of possible base pairs as they are always presented by the unmodified RNA sequence. All necessary corrections due to the base modifications are performed by the constraints framework that guarantees that loops are treated differently if (i) they consist of at least one modified bases and (ii) corresponding energy parameters or changes in pairing partner preference are available. In these cases, an energy correction term (soft constraint) is applied to compensate for the error of treating the loop as if it was unmodified. Any changes in base pairing partner preference due to the modification are handled as hard constraints. See Supplementary Section S1 for further details.

### 2.2 Energy parameters

At the time of writing, our literature search on available NN energy parameters that include modified bases is limited to only a handful of datasets, most of them derived from UV-melting experiments. Richardson and Znosko (2016) list base pair stacking and helix-end parameters for *7-deaza-adenosine* • *uridine* base pairs. In Wright *et al.* (2007) and Wright *et al.* (2018), stacking and helix-end parameters are reported for *inosine* • *uridine* and *inosine* • *cytosine* pairs, respectively. Kierzek *et al.* (2022) list parameters for *N6-methyladenosine* • *uridine* pairs, in particular base pair stacking, helix-end, and terminal mismatch energies. *Pseudouridine* • *adenosine* stacking and helix-end parameters can be taken from Hudson *et al.* (2013). The non-standard nucleotide purine, also known as nebularine, is able to pair with uridine. For this modified base, stacking and helix-end parameters are available in Jolley and Znosko (2017). So far, no parameters have been determined for *dihydrouridine*. However, Dalluge *et al.* (1996) performed NMR spectroscopy experiments on dihydrouridine mononucleotides and very short oligos to find that this type of modification promotes the C2’-endo sugar conformation, thereby destabilizing potential stacking interactions. They determined ΔH values of 1.5–5.3 kcal⋅mol^−1^ that quantify this destabilizing effect. Chou *et al.* (2016) proposed a framework based on Monte-Carlo simulation of small structure motifs to predict NN parameters. Following this approach, we simulated NN parameters for *dihydrouridine* • *adenosine* base pairs and found destabilizing effects of up to 1.4 kcal⋅mol^−1^ in terms of free energy, suggesting that the *in silico* estimates are reasonable. Details of these computations can be found in Supplementary Section S3.

Note, that virtually all of the above parameter sets are incomplete in the sense that particular stacking bases have not been measured or reported, e.g. pairs with a modified base followed by a *G* • *U* wobble pair. Other NN parameters in these sets, for instance terminal mismatches or dangling end contributions, only represent a small subset of possibilities.

We gathered the above mentioned parameter sets and include them in the ViennaRNA Package as JSON-formatted files. Our software provides the means to load these files and automatically prepare the constraints framework according to the specifications stored in them. This allows to easily extend the list of modified bases as soon as new energy parameters become available by simply creating additional files. At the same time, this makes the parameter sets available for third-party applications. Currently, our implementations allow to load NN parameters for: (i) base pair stacks, (ii) helix-end, (iii) terminal mismatches, and (iv) dangling ends. Each of the parameter sets must list free energy values $\Delta G_{37}$ measured at T=310.15 K (37°C) and 1 M NaCl to maintain compatibility with the remaining NN parameters. In addition, enthalpies ΔH can be provided to allow for rescaling of the free energies $\Delta G_{T}=\Delta H-T\Delta S$ at temperatures *T* other than 37°C. The JSON data structure for each modified base further specifies one-letter-codes for (i) the modified base, (ii) its unmodified counterpart, as well as (iii) a fallback base used whenever no additional parameters are available. A detailed specification and description of the format is given in the Supplementary Section S2.

### 2.3 Inclusion into the ViennaRNA Package

The approach to include modified bases by means of constraints enabled us to adapt multiple structure prediction algorithms with only little effort. As a first step, the programs RNAfold and RNAsubopt are now capable to predict global structures and base pairing probabilities, as well as suboptimal structures for input sequences with modified bases, respectively. For local structure prediction and accessibility computations, the programs RNALfold and RNAplfold have been adapted accordingly. Finally, introducing the new feature to RNAcofold allows predicting the structures of two interacting RNAs, where one or both sequences contain modified bases. All the above programs are capable to parse input sequences containing the one-letter-code of the respective modified base. Along with that we adapted our RNAfold WebService (http://rna.tbi.univie.ac.at) to also expose the new feature and to allow for pre-processing of input sequences with base modifications, see also suppementary section S1.7. Energy corrections for the modified bases mentioned in Section 2.2 are already compiled into the programs to avoid the requirement to load additional parameter files. The new -m command line option activates support for the built-in modified bases. In addition, the programs can be provided further parameters from JSON files through the command line option—mod-file, see also Supplementary Section S1.6 and the man pages of the respective programs. In terms of runtime we observe no change in runtime for unmodified and a moderate impact for sequences with only a few modified bases. However, for heavily modified sequences, e.g. tRNAs, the constraint evaluations may be more noticable. The tRNAdb lists 623 tRNA sequences with an average length of 77.26 nt and an average of 8.82 modifications per sequence. On an Intel(R) Core i7-9700K machine running at 3.6 GHz with Fedora Linux 37 the total time for RNAfold to predict MFE structures for all sequences increases from about 1.35 to 1.95 s, which corresponds to a slowdown of a factor of 1.45. The total overhead when including partition function and base pair probability computation amounts to about 90%.

The low-level constraint framework for modified bases is also available through the ViennaRNA C-library RNAlib. Both approaches, reading parameters from JSON files as well as using the compiled-in parameter sets are exposed as dedicated functions in the library. This enables third-party tools to easily adapt the prediction made with RNAlib to additionally support modified base corrections. Finally, this functionality is also exposed through the scripting language interfaces of RNAlib, making them available to be readily included in Python or Perl scripts and pipelines.

## 3 Results

**Figure 1.**
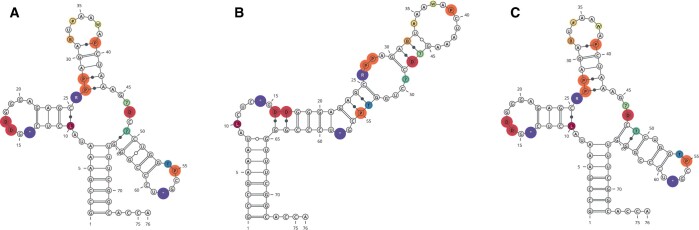
The structure of *Bos taurus* tRNA-Phe (tRNAdb ID: tdbR00000096) (Jühling *et al.* 2009). (A) Reference structure, (B) MFE prediction for unmodified sequence, and (C) MFE prediction using RNAfold with modified base support. Positions of modified bases are highlighted with colors, where larger circles indicate the presence of additional energy parameters and pairing rules [*pseudouridine* (P)—orange, *dihydrouridine* (D)—red, as well as non-paring $m^{1}A$ (”) and $m_{2}^{2}G$ (R)—both purple]. Positions marked by small circles denote modified bases where no additional rules are available, and for which unmodified versions were used in a prediction. Structure layout plots have been created with VARNA (Darty *et al.* 2009).

As an example, we predicted the secondary structure of *Bos taurus* tRNA-Phe (ID: tdbR00000096) as available from tRNAdb (Jühling *et al.* 2009). This RNA contains 17 modified nucleotides (see Fig. 1A). For most of these modifications (*m*^2^*G*, *Cm*, *Gm*, *o2yW*, *m*^7^*G*, *m*^5^*C*, and *m*^5^*U* with tRNAdb one-letter codes L, B, #, W, 7, ?, and T, resp.), the effect on secondary structure is unknown, so we simply treated them as if they were unmodified. Conversely, energy corrections are included in our tools for *pseudouridine* (P) and *dihydrouridine* (D). In addition, we identified modifications that are known to prevent base pairing (Motorin *et al.* 2007): $m^{1}A$ (tRNAdb symbol “), and $m_{2}^{2}G$ (tRNAdb symbol R). Consequently, we prevent those from pairing using hard constraints.

To assess the effect of taking into account the additional data on modified bases, we performed two predictions. We first predicted the secondary structure of the sequence without providing any data for modified nucleotides. In this case, we replaced all modified nucleotides by their unmodified counterpart for the computation, e.g. $m^{1}A$ by *A*, *P* by *U*, etc. The resulting minimum free energy (MFE) structure does not resemble the expected tRNA cloverleaf (Fig. 1B). In fact, it has a base pair distance of 29 to the accepted reference structure, see also Supplementary Section S4. A more detailed analysis of the structure ensemble reveals that the majority of structures are paired differently compared to the reference structure. The ensemble defect (Dirks *et al.* 2004) with respect to the reference is 0.46, meaning that on average about half of the nucleotides (46%) do not pair as proposed by the reference cloverleaf structure. In contrast, when the (available) energy contributions of modified bases and their change in pairing behavior is taken into account, the predictions are very close to the reference structure (Fig. 1C). The predicted MFE structure still has a base pair distance of 10 from the reference, due to a shift by one nucleotide in the TΨC-arm. However, suboptimal structure prediction shows the reference structure as second-most stable with an energy difference of only 0.2 kcal⋅mol^−1^ to the ground state. The two structures would thus have similar occupancy in equilibrium. Moreover, including the modified base corrections drastically changes the structure ensemble toward the accepted cloverleaf fold, as expressed by the ensemble defect of just 0.16. A visual comparison of the predicted base pair probabilities with and without modified base support can be found in Supplementary Fig. S2.

## 4 Conclusion

We have shown how to add modified base support to existing RNA secondary structure prediction algorithms without the need to modify or rewrite the core prediction algorithms. Our plugin-like constraints framework automatically corrects for changes in energy contributions and pairing partner preferences induced by the base modification and as specified in a provided set of parameters. In contrast to RNAstructure, the only other RNA secondary structure prediction tool capable to support modified bases we are aware of, our approach does not require the user to compile a complete set of NN energy parameters. Instead, only parameters for loops with modified bases that are actually available have to be fed into our algorithms. The JSON format we have chosen is simple and files for new parameter sets can be generated with little effort. At the same time it is easily extendible to support more loop types in the future when such parameters become available from either experiments or predictions.

The constraints framework to support modified bases, at this time, always assumes that modified bases can only interact to form base pairs with nucleotides that are either unmodified or of the same type. Consequently, our current approach lacks support for two distinct modified bases pairing with each other. When the prediction algorithms evaluate a loop with two different modified bases forming a base pair, its total contribution becomes the sum of differences to the unmodified case, see Supplementary Section S1.1. We are aware that this crude estimate may be wrong, but currently, no NN energy parameters where two distinct modified bases form base pairs are available. Since such data may become available in the future, we will adapt the implementations accordingly in one of the next releases of the ViennaRNA Package. In addition, we will address the implementation of the modified base support to the remaining algorithms included in our software, such as consensus structure prediction with RNAalifold and RNALalifold, as well as the RNA-RNA interaction predictions performed by RNAmultifold.

As of version 2.6.0, the ViennaRNA Package includes modified base data for $m^{6}A$, *inosine*, *pseudouridine*, *dihydrouridine*, *7DA*, and *nebularine*. However, there are many more modifications for which parameters are not yet available, and research on the effects of those modifications is ongoing. In addition, the experimental identification of modified bases, their positions within a sequence and their actual chemical nature is still a difficult task. But recent advances in RNA sequencing may hold the key to eventually unlock a much larger amount of annotated modified bases for various RNA sequences (Harcourt *et al.* 2017, Leger *et al.* 2021). Adding support for modified bases to the prediction algorithms can drastically change the predicted structures. This can not only be observed for our tRNA-Phe example but applies more generally. MFE prediction performances for all 623 tRNA sequences in tRNAdb show a substantial increase when modified bases are taken into account, see Supplementary Section 4.3. Consequently, (tertiary) structure models that build upon secondary structures may benefit from the additional data modified bases provide. Given the necessity of understanding the influence of modifications on RNA functionality and the increasing number of studies dedicated to the topic, we expect additional data to become available in the future. This data would be important for improving structure prediction methods and potentially lead to a better understanding of the relationship between structure and function of various RNAs.

## Supplementary Material

btad696_Supplementary_Data
