# Supplementary material for: Modified RNAs and predictions with the ViennaRNA Package
Source: Bioinformatics. 2023 Nov 16;39(11):btad696. doi: 10.1093/bioinformatics/btad696 (PMC10676514; doi:10.1093/bioinformatics/btad696)
Supplement: btad696_Supplementary_Data [file btad696_supplementary_data.pdf]

Modified RNAs and Predictions with the ViennaRNA Package

Supplementary Data

Yuliia Varenyk, Thomas Spicher, Ivo L. Hofacker, and Ronny Lorenz

S1 Algorithmic details

In the Nearest Neighbor (NN) energy model (Turner and Mathews, 2009), the total free energy $E(s)$ of a secondary structure s is approximated by the sum of free energies E_L of the loops L s is composed of. i.e.

$$E(s) \approx \sum_{L \in s} E_L \tag{1}$$

Under the presence of energy parameters E_L^m for modified bases, E_L in the above equation has to be substituted by the corresponding parameter E_L^m for loops L that consist of a modified base.

S1.1 Energy Corrections

To accommodate for the effect of modified bases, our approach uses the soft constraints framework (Lorenz et al., 2016) to adjust energy evaluations E_L whenever a modified base is part of L and additional parameters E_L^m are available. To this end, we compute a correction term $\Delta\Delta E_L^m = E_L^m - E_L$ which is added by the soft constraints framework, whenever L is evaluated. Loops without modified bases or those where no additional parameters E_L^m are available yield $\Delta\Delta E_L^m = 0$. Hence, the total free energy given a (sub)set of modified base energy parameters becomes

$$E(s) = \sum_{L \in s} E_L + \Delta\Delta E_L^m. \tag{2}$$

For multiple distinct modifications $m_1, m_2, \ldots, m_i$ with corresponding energy parameters $E_L^{m_i}$ the correction term becomes

$$\Delta\Delta E_L^m = \sum_i \Delta\Delta E_L^{m_i}. \tag{3}$$

Note, that this approach does not allow for precise energy corrections where multiple distinct modified bases are present within the same loop, even if corresponding parameters were available. This is due to the fact that individual $\Delta\Delta E_L^m$ are always differences to the standard NN energy parameters without modified bases.

S1.2 Fallback Bases

Available energy parameters for modified bases are sparse and most often restricted to those for stacking base pairs. Within our approach, any loop L that contains a modified base but where a corresponding E_L^m is unavailable, will be treated as if all bases were unmodified. However, simply using the unmodified equivalent of a modified base may be the wrong choice. For instance, inosine is known to behave more similar to guanosine than its unmodified counterpart adenosine, although stacks containing $I \bullet C$ base pairs are generally less stable

than those with $G \bullet C$ (Wright et al., 2018). So it makes sense to treat all inosines as if they were guanosines whenever no specific parameter E_L^m is present. For that purpose, we introduce a *fallback* for each modified base that can be specified in the parameter set. In the simplest case, the fallback is the unmodified equivalent, but it can be any of the four canonical RNA bases ACGU.

S1.3 Base Pairing Partner Preferences

Some modifications change the preferred base pairing partners relative to their unmodified equivalent. Inosine, for instance, forms almost iso-sterical base pairs with cytosine and uridine, while the unmodified adenine only forms iso-sterical base pairs with uridine. Our approach, therefore, allows one to specify a set of base pairing partners for the modified base. Internally, the hard constraints framework then changes the recursive algorithms such that only those loops are evaluated where the base pairing partner preferences are met.

In cases where a modified base can not form iso-sterical base pairs at all, e.g. due to a methylation at its Watson-Crick side, the modified base should be separately forced to stay unpaired via the constraints interface.

S1.4 Nearest Neighbor Energy Parameter Tables

The parameters for the NN energy model are typically stored as multi-dimensional lookup tables, where each dimension corresponds to one of the delimiters of a loop, e.g. one or two base pairs and sometimes a small number of associated individual bases. Stacked base pairs, for instance, require a 2-dimensional matrix of size $N \times N$, where N is the number of possible base pairs, reflecting each combination of one base pair stacking onto any other base pair. While most energy tables are 2–3 dimensional, the most extreme case in the NN model are internal loops with 2 unpaired bases within the first and 3 unpaired bases in the second part of the loop. Adding the two base pairs that enclose such an internal loop yields an energy table of size $N^2 \times |\Sigma|^5$ where $|\Sigma|$ is the size of the nucleotide alphabet. Apart from the vast amount of experiments required to determine those parameters, extending the NN tables by more than just as handful of modified bases results in very memory consuming data structures.

In particular, for each modified base $|\Sigma|$ increments by 1 while N grows by at least 2. This is due to directedness of the RNA strand from $5'$ to $3'$ and the resulting non-symmetric nature of the energy parameters. Additionally, if a (modified) base has multiple potential pairing partners, N increases by 2 per pairing partner.

S1.5 Availability in the `ViennaRNA Package` API

Our implementations for the modified base support are made available in the `RNAlib` C-library, that comes with the `ViennaRNA Package`. The declarations can be included via the C-header file

```
#include <ViennaRNA/constraints/soft_special.h>
```

Among the declared functions are those that load and prepare JSON formatted parameters. They generate a data structure suitable to be passed to the modified bases soft constraint generator, which in turn takes care of all preparations for subsequent structure predictions. In addition, we provide high-level functions to automatically load the sets of built-in energy parameters, where only the sequence positions of the modified bases have to be provided. All of the above functions are also exposed through out scripting language interfaces. This makes them easily available for Perl 5 and Python scripts and pipelines.

For specific details and code examples, we refer to the ViennaRNA reference manual available at https://viennarna.readthedocs.io.

S1.6 Availability in executable ViennaRNA Package programs

We added two command line flags to a series of executable programs shipped with the ViennaRNA Package to allow our users to easily activate modified base support in the predictions algorithms. The flag -m, --modifications[=STRING] activates support for the build-in modified base energy parameters. The modified bases in the input sequence(s) must be marked by their corresponding one-letter code. If the optional argument STRING is omitted, all available corrections are performed. Otherwise, users may limit the type of supported modifications to a particular subset, resp. one-letter codes, by specifying them in STRING. For instance, --modifications="P6" will only correct for pseudouridine and m6A bases. Currently supported one-letter codes and energy corrections can be found in Table S1.

One-letter-code	Modified base
7	7-deaza-adenonsine (7DA)
I	Inosine
6	N6-methyladenosine (m^6A)
P	Pseudouridine
9	Purine (a.k.a. nebularine)
D	Dihydrouridine

Table S1: Modified bases currently supported by our implementations. Shown are one-letter codes to be used in the input sequence for our executable programs and their corresponding (trivial) names. Energy parameter files are distributed with the ViennaRNA Package for each of them.

Additional modified base parameters can be loaded using the parameter --mod-file=<filename>, where <filename> is a JSON file that specifies metadata and the energy parameters of the modified base, see also Section S2 below. This parameter can be specified multiple times, thus many different parameter sets can be supplied at the same time.

At the time of writing, the following executable programs received modified base support: `RNAfold`, `RNALfold`, `RNAplfold`, `RNAsubopt`, and `RNAcofold`. Further support in other programs shipped with the `ViennaRNA Package` will be provided in future releases.

S1.7 Availability in the `ViennaRNA Package` WebServices

We provide online access to the most widely used programs of the `ViennaRNA Package` at `http://rna.tbi.univie.ac.at`. To allow our users to make use of the new modified base support we adapted the `RNAfold` WebService to expose the option to take base modifications into account. In addition, this WebService now integrates an input sequence pre-processing step to handle `tRNAdb` one-letter-codes. In particular, one-letter-code can be translated back into the corresponding unmodified bases (while keeping those modifications that are already supported by `RNAfold`) and modifications known to block reverse transcriptase (RT) (Motorin et al., 2007) that can be assumed to prevent base pairing can be translated into the respective structure constraints.

S2 Energy Parameter specifications

To supply our implementations with energy parameters for modified bases we specify a `JSON` file format. This not only renders the corresponding data machine-readable but, at the same time, is fairly easy to extend or generate for any new sets of parameters. Currently, `JSON` files must not list parameters for more than one modified base. This limitation will be lifted in future releases of the `ViennaRNA Package`.

Each parameter set is represented by an object `"modified_base":{}` whose keys specify energy parameters and meta data for the modified base. Meta data consists of a trivial name, an array of data sources, upper-case one-letter-codes ($A, B, C, D, \ldots$) for the modified base (`"one_letter_code": "A"`), its unmodified counterpart (`"unmodified": "B"`), a fallback base (`"fallback": "C"`), and an array of base pairing partners (`"pairing_partners": ["D", "..." ]`), see also lines 3–18 in Figure S1.

Energy parameters are supplied as additional objects that separately store the free energy (ΔG_{37}) and/or enthalpy (ΔH) values. The corresponding keys for these objects have suffixes `_energies` and `_enthalpies`, respectively. The following key prefixes are currently recognized: (i) `stacking` for base pair stacking data, (ii) `terminal` for helix end contributions, (iii) `mismatch` for terminal mismatches, (iv) `dangle5` for $5'$ dangles, and (v) `dangle3` for $3'$ dangles. Each such energy parameter object then consists of a set of key-value pairs where the keys correspond to the sequence motif that delimits the loop, and the values are energies in units of $kcal \cdot mol^{-1}$ measured at 1 M NaCl. Free energies have to correspond to $T = 310.15\,\text{K}$, i.e. $37\,^{\circ}\text{C}$. Notably, keys for stacked pairs are strings of length 4 where the first two characters are the one-letter-codes of bases on the first strand segment in $5'$ to $3'$ direction, and the last two characters are

the pairing partners in $3'$ to $5'$ direction. The same order holds for terminal mismatch keys, where the first and third characters denote the base pair and the second and fourth characters denote the $3'$ and $5'$ mismatching bases, respectively. Keys for dangling end contributions are of length 3 where the first two characters denote the base pair and the third character is the single nucleotide that stacks onto it, either from $5'$ or $3'$ side. All keys may consist of the characters $\Sigma = \{A, C, G, U, M\}$ where M is the one-letter-code of the modified base as specified in the `"one_letter_code":""` string. Figure S1 shows a `JSON` template for an artificial modified base with one-letter-code M that provides at least one parameter for each of the currently recognized loop types.

All energy parameter objects are optional and can be left out from the parameter set in the `JSON` file. When enthalpies ΔH are given, corresponding free energies ΔG_{37} must be provided as well. Such parameter pairs are then subject to free energy rescaling

$$\Delta G_T = \Delta H - T\Delta S = \Delta H - \frac{T}{310.15\,K}(\Delta H - \Delta G_{37}) \tag{4}$$

whenever the prediction temperature T deviates from the default $T = 310.15\,\mathrm{K}$.

S3 In silico estimation of dihydrouridine stacking energies

Chou et al. (2016) developed a framework to use coarse grained RNA folding simulations of small RNA duplexes to extract the NN parameters from the `Rosetta` energy function. In previous analyses, this `RECCES` framework has shown the capacity of predicting some NN parameters with an accuracy comparable to experimental results. Here, we use this in silico approach to estimate the NN free energy parameters for stacked pairs that contain dihydrouridine (D), i.e. stacks of D $\bullet$ A base pairs.

For that purpose, we simulated the following duplexes:

```
5'GGD  5'GAU  5'GUA  5'GAC  5'GAG
3'CCA  3'CDA  3'CAD  3'CDG  3'CDC

5'GDA  5'GCD  5'GAA  5'GDC  5'GUD
3'CAU  3'CGA  3'CDU  3'CAG  3'CAA

5'GAD  5'GGA  5'GDG  5'GDU  5'GCA
3'CUA  3'CCD  3'CAC  3'CAA  3'CGD

5'GC  5'GA  5'GD  5'GG  5'GA  5'GU
3'CG  3'CU  3'CA  3'CC  3'CD  3'CA
```

In equation 5 we show the set-up to get the energy contribution for each stack (ΔG_{NN}) from the different simulations (ΔG_f). Furthermore, this shows how one can get rid of the so-called AU penalty term $\Delta G_{\text{Term-AU}}$ for $A \bullet U$ base pairs at the end of helices which is treated as a separate term in the NN energy model.

```json
{
  "modified_base" : {
    "name" : "My modification (M)",
    "sources" : [
      {
        "authors" : "Author 1, Author 2",
        "title" : "UV-melting of modified oligos",
        "journal" : "Some journal",
        "year" : 2022,
        "doi" : "10.0000/000000"
      }
    ],
    "unmodified" : "G",
    "pairing_partners" : [
      "U","A"
    ],
    "one_letter_code" : "M",
    "fallback" : "G",
    "stacking_energies" : {
      "MAUU" :  -1.2,
      "AGMC" :  -2.73
    },
    "stacking_enthalpies" : {
      "MAUU" :  -11.1,
      "AGMC" :  -9.73
    },
    "terminal_energies" : {
      "MU" : 0.5,
      "UM" : 0.5
    },
    "terminal_enthalpies" : {
      "MU" : 2.0,
      "UM" : 2.0
    },
    "mismatch_energies" : {
      "CMGM" : -1.11,
      "AGUM" : -0.73
    },
    "mismatch_enthalpies" : {
      "CMGM" : -11.11,
      "AGUM" : -7.73
    },
    "dangle5_energies" : {
      "UAM" : -1.01
    },
    "dangle5_enthalpies" : {
      "UAM" : -6.01
    },
    "dangle3_energies" : {
      "CGM" : -2.1,
      "GCM" : -1.3
    }
  }
}
```

Figure S1: **A JSON template for energy parameters with modified bases.** Here, the modified base has one-letter-code M, is derived from a G and may pair with either U or A.

$$\begin{aligned}\Delta G_{NN}\binom{\text{5'AX}}{\text{3'DY}} =& \Delta G_{NN}\binom{\text{5'YD}}{\text{3'XA}} \\ =& \frac{1}{2}\Big[\Delta G_f\binom{\text{5'GAX}}{\text{3'CDY}} - \Delta G_{\text{Term-XY}} \\ & - \Delta G_f\binom{\text{5'GA}}{\text{3'CD}} + \Delta G_{\text{Term-AD}} \\ & + \Delta G_f\binom{\text{5'GYD}}{\text{3'CXA}} - \Delta G_{\text{Term-AD}} \\ & - \Delta G_f\binom{\text{5'GY}}{\text{3'CX}} + \Delta G_{\text{Term-XY}}\Big]\end{aligned} \tag{5}$$

$$\begin{aligned}\Delta G_{\text{Term-AD}} =& \frac{1}{2}\Big[\Delta G_f\binom{\text{5'GYD}}{\text{3'CXA}} - \Delta G_f\binom{\text{5'GY}}{\text{3'CX}} \\ & - \Delta G_f\binom{\text{5'GAX}}{\text{3'CDY}} + \Delta G_f\binom{\text{5'GA}}{\text{3'CD}}\Big]\end{aligned} \tag{6}$$

Here, $X \bullet Y$ can be any of the unmodified canonical base pairs. The correcting terminal XY and terminal AD terms as required by the NN model cancel each other out. $\Delta G_{NN}\binom{\text{5'DX}}{\text{3'AY}}$ stacks can be computed analogously.

Compared to the original setup in Chou et al. (2016) we adapted our simulations using a moderate increase of the length of the MC simulations. The pre-run to determine the parameters regulating the temperature switches during the simulated tempering was increased from $300,000$ to $400,000$ steps. The simulated tempering steps themselves were also increased from $9,000,000$ to $12,000,000$. This reduced the standard deviation between the results of the different simulation runs. Next to the stacking energies we also computed the terminal penalty $\Delta G_{\text{Term-AD}}$ according to equation (6), see the supplement part of Chou et al. (2016) for further details. The results of our simulations can be found in Table S2.

Furthermore, we used version 3.13 of the `Rosetta` software package, while the original publication was based on version 3.6.

During our simulations with the `Rosetta` software we identified two bugs that resulted in erroneous simulation results for most modified bases. The first bug resulted from incorrect sampling of sugar pucker conformations, and the second gave wrong results unless the duplex started with a $G \bullet C$ pair. Fixes for the bugs were communicated to the upstream developers and found their way in recent versions of `Rosetta` software.

	**RECCES**	**stdv**	$\Delta\Delta G_{NN}(A \bullet U)$	ΔH	**stdv**
5'AU<br>3'DA	−0.62	0.09	0.48	−7.65	0.40
5'AG<br>3'DC	−2.03	0.07	0.05	−9.80	0.29
5'AC<br>3'DG	−1.00	0.06	1.24	−7.38	0.22
5'AA<br>3'DU	−0.54	0.09	0.39	−8.07	0.32
5'UA<br>3'AD	−0.49	0.07	0.84	−9.44	0.21
5'GA<br>3'CD	−1.28	0.07	1.07	−10.55	0.33
5'CA<br>3'GD	−0.71	0.05	1.40	−9.10	0.27
5'AA<br>3'UD	−0.61	0.07	0.32	−8.57	0.30
AD Term.	0.26	0.05		0.44	0.26

Table S2: NN parameters containing dihydrouridine-adenosine base pair. Calculation with the RECCES method in the first column, and the standard deviation (stdv) from 4 independent simulations in the second. These predictions are compared to the corresponding $A \bullet U$ stacks ($\Delta\Delta G_{NN}(A \bullet U)$). The two last columns show the enthalpies (ΔH) and the corresponding standard deviation extracted from the RECCES simulations.

S4 tRNA structure prediction with modified bases

As an example, we used tRNA-Phe from *Bos taurus* with a total of 17 annotated modified bases and `tRNAdb` ID tdbR00000096 (Jühling et al., 2009):

```
>tdbR00000096 tRNA-Phe Bos taurus anticodon #AA
GCCGAAAUALCUC"GDDGGGAGAGCRPPAGABU#AAWAPCUAAAG7DC?CUGGTPCG"UCCCGGGUUUCGGCACCA
(((((((..((((........)))).(((((.......))))).....(((((.......))))))))))))....
```

The one-letter codes and their corresponding modified base as used in the tRNA sequence can be taken from Table S3. We then predicted MFE structures for this sequence with and without modified base support to compare the results against the annotated reference structure as obtained from `tRNAdb`. For the latter, all base modifications that are currently not recognized by our implementations were replaced by their unmodified counterparts. In addition, base modifications that are known to prevent base pairing (Motorin et al., 2007) were forced to stay unpaired using hard constraints. In addition, we use the following two metrics to quantify the distance between the prediction and the reference structure.

Symbol	Modified nucleotide	Corrections
P	*pseudouridine*	build-in
D	*dihydrouridine*	build-in
"	m^1A	hard constraints
R	m_2^2G	hard constraints
L	m^2G	-
B	Cm	-
#	Gm	-
W	*peroxywybutosine*	-
7	m^7G	-
?	m^5C	-
T	m^5U	-

Table S3: Symbols for modified nucleotides occurring in sequence tdbR00000096 per `tRNAdb` nomenclature (Jühling et al., 2009). Column **Corrections** denotes whether (i) our prediction tools corrected for the presence of this modification via the new modified base support (*build-in*), (ii) we applied additional hard constraints (*hard constraints*) to prevent this base from pairing, or (iii) the modification was substituted by the corresponding unmodified base (-).

S4.1 Base Pair Distance

A simple metric for secondary structures over the same sequence is the base pair distance

$$d_{\mathrm{BP}}(s,t) = |s \cup t| - |s \cap t| \tag{7}$$

between any two secondary structures s and t. This distance simply counts the number of base pairs both structures do not have in common.

S4.2 Ensemble Defect

Instead of measuring the distance from s to another individual secondary structures t, a more comprehensive overview over the underlying structure ensemble can be gained from the ensemble defect

$$\mathrm{ED}(s) = \frac{1}{n}\sum_{t} p(t) 2 d_{\mathrm{BP}}(s,t) \tag{8}$$

with respect to s. This measures the expected fraction of nucleotides that are paired differently compared to s in a structure t randomly drawn from the ensemble. Note, that here t is drawn with equilibrium probability

$$p(t) = \exp(-E(t)/RT)/Z \quad \text{with} \qquad Z = \sum_{s} \exp(-E(s)/RT) \tag{9}$$

where Z is the partition function, $E(t)$ the energy of structure t, T is the thermodynamic temperature and gas constant R.

S4.3 Results

The MFE structures s_{MFE} obtained from predictions using sequences with and without modified bases do not fully resemble the tRNA cloverleaf reference structure s_{ref}. As expected, the difference is the largest for the fully unmodified sequence that shows a base pair distance $d_{\mathrm{BP}} = 29$, i.e. to transform the MFE structure into the reference a total of 29 base pairs have to be removed or added. The ensemble defect of 46 % tells us that for the majority of the structure ensemble about half of their nucleotides are paired differently compared to the reference structure. As soon as we apply the novel modified base support and exclude those nucleotides from base pairing that are known to block reverse transcriptase, the predictions become much closer to the reference. The base pair distance of the MFE structure to the reference drops to just 10 and the ensemble defect drops by 30 % to only 0.16, see also Table S4. A detailed analysis of the energies for all near ground state structures reveals that the actual reference structure is only $0.2\, kcal \cdot mol^{-1}$ from the predicted MFE when using modified base support. A comparison of the predicted pairing probabilities for base pairs (i, j)

$$p_{ij} = \frac{1}{Z} \sum_{s|(i,j)\in s} e^{-E(s)/RT} \tag{10}$$

with and without modified base support clearly shows the shift of the ensemble towards the correct cloverleaf conformation, see Figure S2.

	$d_{\mathrm{BP}}(s_{\mathrm{MFE}}, s_{\mathrm{ref}})$	$\mathrm{ED}(s_{\mathrm{ref}})$	$E(s_{\mathrm{ref}})$	$E(s_{\mathrm{MFE}})$
w/o modified bases	29	0.46	-21.90	-23.60
modified base support	10	0.16	-22.27	-22.47

Table S4: Calculations of base pair distance d_{BP}, ensemble defect $ED(s)$, and energies $E(s)$ of the reference- and MFE structures (s_{ref}, s_{MFE}) with and without modified bases support in `ViennaRNA Package`. Decrease in base pair distance and ensemble defect measures (wrt. the reference structure) after introducing parameters for modified bases indicates a significant shift of the ensemble towards the reference structure. Energies $E(s)$ are given in units of $kcal \cdot mol^{-1}$.

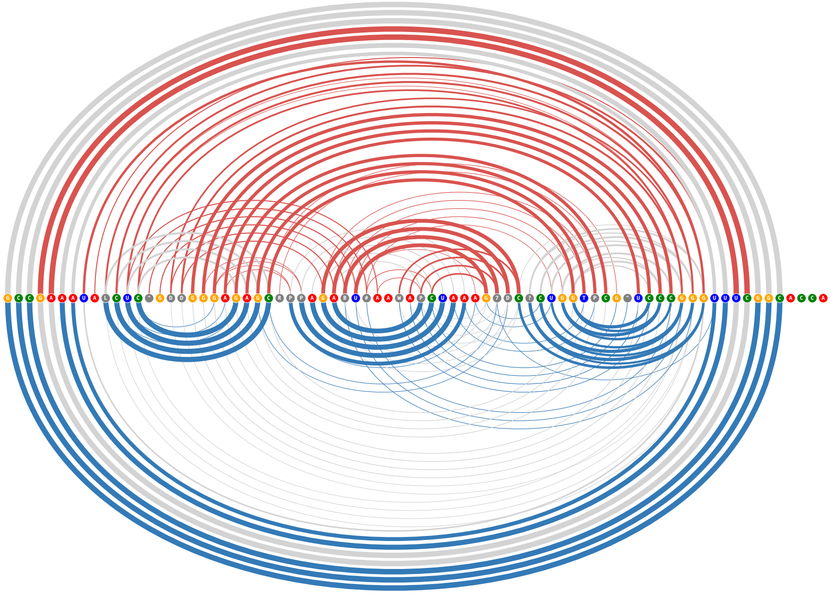

Figure S2: **Base pair probabilities with and without modified base support**. Here, base pair probabilities for the unmodified *Bos Taurus* tRNA-Phe (*ID tdbR00000096*) sequence and the one with supported modifications are shown as arcs on the top and bottom, respectively. The linewidth of the arcs is proportional to the corresponding probability and arcs appear colored (*red for unmodified, blue for modified sequence*) if the probability under the respective model is larger than for the other model. Otherwise, arcs appear in grey. Modified bases are depicted as grey circles.

We further predicted MFE structures for the entire `tRNAdb` data set. At the time of writing this set consist of 623 sequences. Among the modified bases considered in our predictions are those that are known to block reverse transcriptase (RT): m^1A, m^1G, m^2_2G, m^3U, $m^3\Psi$, acp^3U, m^3C, and *queuosine* (Motorin

et al., 2007). These bases were effectively constrained to stay unpaired in the predictions. The only remaining modified bases that we currently have energy parameters for are *dihydrouridine* (D) and *pseudouridine* (P). For those our implementations applied energy corrections. To quantify the differences in prediction performance with and without modified base support, we computed (i) the *true positive rate* (TPR), a.k.a. *sensitivity*,

$$\mathrm{TPR} = \frac{\mathrm{TP}}{\mathrm{TP} + \mathrm{FN}} \tag{11}$$

that measures the proportion of correctly predicted base pairs from the reference structure and (ii) the positive predictive value (PPV)

$$\mathrm{PPV} = \frac{\mathrm{TP}}{\mathrm{TP} + \mathrm{FP}} \tag{12}$$

to assess the proportion of correctly predicted base pairs among the total number of predicted base pairs. For that purpose, we extracted from our predictions the number of *true positives* (TP), i.e. base pairs that are actually part of the reference structure, *false negatives* (FN), i.e. base pairs that are part of the reference structure but missing in the prediction, as well as *false positives*, i.e. those base pairs that are predicted but not part of the reference. These values then underwent a bootstrapping analysis with $1,000$ iterations to obtain the 95 % confidence intervals to estimate the robustness of our predictions. As can be seen in Figure S3, the implemented modified base support substantially increases the prediction performance both in terms of more TP and less FP for the known tRNA sequences.

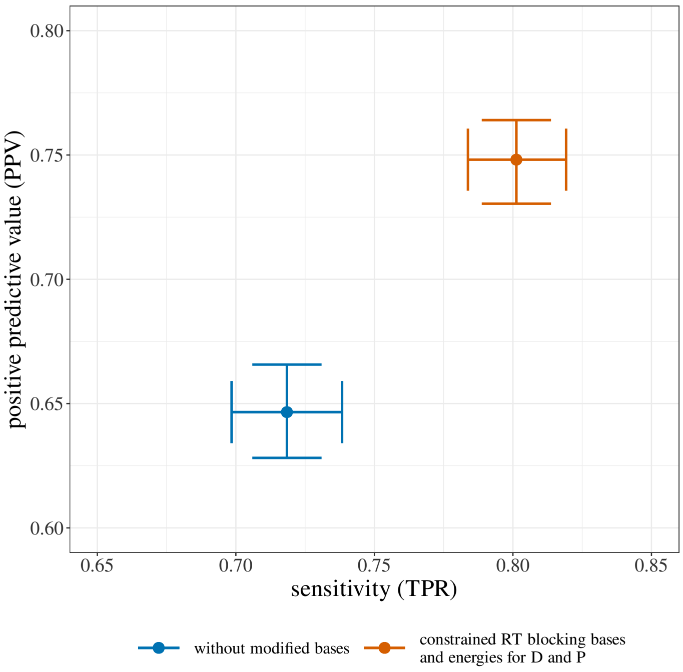

Figure S3: **Prediction performance for the `tRNAdb` data set**. Shown are the average true positive rate (TPR) and positive predictive value of MFE predictions with and without modified base support for the entire set of 623 tRNAs stored in `tRNAdb`. Error bars indicate 95 % confidence intervals estimated from bootstrapping with 1,000 iterations.

References

Chou, F.-C. et al. (2016). Blind tests of RNA nearest-neighbor energy prediction. Proceedings of the National Academy of Sciences, **113**(30), 8430–8435.

Jühling, F. et al. (2009). tRNAdb 2009: compilation of tRNA sequences and tRNA genes. Nucleic Acids Research, **37**(suppl_1), D159–D162.

Lorenz, R. et al. (2016). RNA folding with hard and soft constraints. Algorithms for Molecular Biology, **11**(1), 1–13.

Motorin, Y. et al. (2007). Identification of modified residues in RNAs by reverse transcription-based methods. Methods in Enzymology, **425**, 21–53.

Turner, D. H. and Mathews, D. H. (2009). NNDB: the nearest neighbor parameter database for predicting stability of nucleic acid secondary structure. Nucleic Acids Research, **38**(suppl_1), D280–282.

Wright, D. J. et al. (2018). Stability of RNA duplexes containing inosine· cytosine pairs. Nucleic Acids Research, **46**(22), 12099–12108.
